# In Memoriam—Dr. Joshua Emmons

**Published:** 1895-01

**Authors:** I. Dever

**Affiliations:** Clinton, N. Y.


### IN MEMORIAM—DR. JOSHUA EMMONS.

Dr. Joshua Emmons died November 26th, 1894, at 1.30 P. M., at his home, No. 17 South Eighth Street, Richmond, Ind.

On the evening of April 6th, he sustained a fracture of the neck of the femur, while alighting from an electric car in motion.

He thought he was able and did resume his practice in July, but the first of September he was compelled to abandon it and confine himself to his room, and finally his bed, where he received the kind and loving care of his devoted wife and son until called from labor to reward.

Dr. Emmons was born near Middletown, Butler County, O., on the 5th day of December, 1826. His youth was spent on the farm, during which time he attended the district school and the Monroe Academy. He taught school at the age of seventeen, and continued teaching as a profession up to the time when he entered the Eclectic Medical Institute, of Cincinnati, from which he graduated in 1850. At the time of his attendance the Institute was provided with one homœopathic professor, under whose teaching the Doctor became imbued with the spirit of Homœopathy. He was a reformer by nature, and notwithstanding we find him, shortly after his graduation, located at Piqua, O., as an eclectic physician and as a member of the Montgomery County Eclectic Society, he presented a paper to the Society advocating the claims of Homœopathy in the treatment of all forms of disease. *Some* of the faithful brothers attempted to bring charges against him for heresy, but as the Society soon became the Montgomery County *Homœopathic* Medical Society, there were none left to "cast the first stone."

Dr. Emmons was an ideal homœopath—a man of extensive reading and deep practical thought. As a prescriber he was one of the first in the homœopathic ranks. He always selected his remedy with care, and prescribed with a precision born of an experimental knowledge which seldom carried short of the mark. Like many thinking physicians, who think out the great truths taught in *The Organon*, he was a Hahnemannian.

Although not obtrusive, he was persistent and faithful in the advancement of the cause which he espoused. He was intensely loyal to his God, to his country, to Homœopathy, and to his friends.

He was an instructive writer and should have written more, as he was the possessor of a vast amount of practical experience with the single remedy in the high and highest powers.

Dr. Emmons located in Richmond, Ind., in 1868, where his gentlemanly deportment and high professional attainments soon introduced him to an extensive practice.

He was thrice married—his first wife being a daughter of the late Dr. Pretsinger, of Euphemia, O. November 5th, 1869, he married Miss Louise M. Bowers, of Richmond, Ind., who proved herself a helpmate in the full sense of the word. She, with a son by the first marriage, are comforted by hope in a future existence and the remembrance of a kind and faithful husband and father, to whose many virtues we may well point as an example to men. I. Dever.

Clinton, N. Y., December 25th, 1894.

